# Supplementary material for: Visual tracking assessment in a soccer-specific virtual environment: A web-based study
Source: PLoS One. 2022 Jun 9;17(6):e0269643. doi: 10.1371/journal.pone.0269643 (PMC9182227; doi:10.1371/journal.pone.0269643)
Supplement: Attachment — Submitted filename: Response to reviewers.pdf [file pone.0269643.s008.pdf]

Dear Editor, Dear Reviewers,

My colleagues and I thank you again very much for your relevant comments and advice on our manuscript entitled “Visual tracking assessment in a soccer-specific virtual environment: a web-based study”. Please find attached the manuscript revised following your comments. The requested responses and additional control analysis have been incorporated into this revised manuscript. The description of data contained in the additional supplementary files has also been added. You will find the changes colored in red in the revised manuscript. All changes are also noted in this rebuttal letter and the lines mentioned refer to the manuscript file with the tracked changes. We also want to inform you that we have removed the web address for the reference 46 (STATS - SportsVU) because it was not functional, and this one was indicated instead:
https://www.statsperform.com/team-performance/football-performance/optical-tracking/

Thank you again for your comments that helped us improve the manuscript.

Kind regards,

Additional Editor Comments:

1. **I kindly ask you to also specify and describe, in the “Supporting information” section of the manuscript, what data are contained in supplementary files S3, S4, and S5.**

Thank you for the reminder. Here are the added descriptions:

- *“S1 File. The mean ratio of successful visual tracking by participants in both experimental conditions.”*
- *“S2 File. The raw dataset (before the data is centered-reduced) used for the clustering of target-players in STRU condition. Each row represents an individual target-player and each column represents a situational feature (columns 2-9) or a visual tracking ratio (columns 10-12).”*
- *“S3 File. The raw dataset (before the data is centered-reduced) used for the clustering of target-players in UNSTRU condition.”*

The supporting files were rearranged in ascending numerical order according to their first appearance in the manuscript:

- S1 File was first cited on line 293.
- S2 File and S3 File were first cited on line 326.
- S4 File and S5 File were first cited on line 352.

2. **Reviewer 2 Point 3a: In your response to this reviewer comment, you present a table showing tracking performance as function of soccer viewing frequency. You state that even though “mean visual tracking performance appeared to increase with the frequency of soccer game viewing, but we assumed that this effect was not significant due to the large standard deviation”. I have plotted the data you presented, and computed 95% confidence intervals from your reported standard deviations and sample sizes. From these, it looks like there**

could be a significant effect, as in a few instances the means and confidence intervals of tracking performance across soccer viewing frequency do not overlap. Whichever the case, I suggest it is always best to verify ones' assumptions when possible. I thus ask you to explicitly test whether a statistical statistically significant effect exists in these data. Further, I believe the reviewer's main question was whether the observed differences between study groups could be explained by soccer viewing frequency. Thus it would be useful to test and report whether the three study groups differed significantly in soccer viewing frequency (e.g. by running a one-way ANOVA on viewing frequency, with sport practice as the between-subjects main effect. If the groups differ in viewing frequency the same way they differ in tracking performance, then this potential confound should be included and discussed in the discussion section of the manuscript. Moreover, when a reviewer brings up a question, it is good practice to address it both in the response letter as well as in the main manuscript, since future readers may have the same question. I thus ask you to incorporate your response to this reviewer comment in the main manuscript. This could be simply a few lines in the methods or the discussion section of the manuscript, where you point out the issue and report the result of your control analyses (once you have performed them).

Thank you for the insightful comment.

This control analysis has been added in the discussion of the manuscript (lines 443-463) :

"It seems likely that people who regularly watch soccer games would also exhibit enhanced visual tracking abilities in situations with soccer-specific trajectories, even if they do not necessarily play soccer themselves. In this regard, participants were asked to indicate how often they watched soccer games on a 5-point Likert scale (Never - Once a year - Once a month - Once a week - Several times a week) in the online form prior to performing the visual tracking task. The results of a two-way mixed ANOVA revealed a significant effect of condition ($F(1,45)=28.170$, $p<0.001$) but no effect of viewing frequency ($F(4,45)=0.653$, $p=0.628$) and no interaction effect between these two factors ($F(4,45)=0.647$, $p=0.631$) on visual tracking ratio. It appears that participants with higher viewing frequency of soccer games did not show better visual tracking performance, whether faced with situations with soccer-specific trajectories or not. In addition, the results of a one-way ANOVA revealed a significant main effect of the group on viewing frequency ($F(2)=18.501$, $p<0.001$). In pairwise comparisons, a higher viewing frequency was observed in SOCC than in TEAM ($p<0.001$), a higher viewing frequency was observed in SOCC than in NoTEAM ($p<0.001$), but no differences were observed between TEAM and NoTEAM ($p=1.000$). Thus, the groups did not differ equally in viewing frequency of soccer games and visual tracking performance. Overall, the difference in visual tracking performance between participants in situations with soccer-specific trajectories cannot be explained solely by whether or not they regularly watch soccer games. What is important seems to rely more on the viewing perspective than on the viewing frequency."

3. **Reviewer 2 point 3b: As above, I ask you to report the result of these control analyses in the main manuscript.**

Thank you for the comment. As requested, the analysis has been added in the manuscript (lines 463-476):

> *"Attentional demands in the field differ depending on the playing position. Differences in visual tracking performance have been observed between frontcourt and backcourt basketball players [16], and between frontward and backwards rugby players [19]. The defender's viewpoint was chosen in this study because most teammates and opponents can be seen within a maximum visual angle of 180°, which increased the crowding of the field of view by the players. With a more advanced viewpoint on the field, the players to be monitored would have been distributed 360° around the observer. A similar defender viewpoint was used in the study by Roca and colleagues (2013) to investigate participants' anticipation when viewing videos of soccer game situations [24]. It can be assumed that defenders would perform better than strikers in the current investigation. It would be interesting to see if playing position would have also influenced tracking performance in this study, but the sample size of soccer players group did not allow for this statistical analysis (3 strikers, 6 midfielders, 6 defenders, and 1 participant did not fill in his/her playing position)."*

4. **Reviewer 2 point 3d: I suggest you should include in the manuscript these considerations and control analyses regarding frame rate and screen resolution. Perhaps you should also specify that by asking participants to position their eyes at a distance from the screen equivalent to its width, the screen should have subtended approximately 53 degrees of visual angle for all participants. For future reference, you should be aware that there exist validated methods to control viewing distance/stimulus size for online experiments, e.g.:**
   - **Li, Q., Joo, S. J., Yeatman, J. D., & Reinecke, K. (2020). Controlling for participants' viewing distance in large-scale, psychophysical online experiments using a virtual chinrest. Scientific Reports, 10(1), 1-11.**
   - **Lago, M. A. (2021). SimplePhy: An open-source tool for quick online perception experiments. Behavior Research Methods, 53(4), 1669-1676**

Thank you for the comment and the references to methods we were not aware of.

The suggestion has been added in the methods section (lines 221-222):

> *"In this way, the screen should have subtended approximately 53 degrees of visual angle for all participants. "*

These considerations and control analyses have been added in the manuscript (lines 493-512):

> *"This instruction was made to ensure standardization of viewing conditions, as visual tracking performance can be influenced by the tracking area size and the speed of targets [54]. For future online investigations, validated methods are now available to control viewing distance and stimulus size [55, 56]. Regarding the stimulus size issue in the current investigation, the task could only be performed in full screen mode. The width and height of the browser viewport of participants have been recorded to*

control the influence of screen dimension on visual tracking performance. The height of the windows varied from 578 to 1137 pixels and the width of windows varied from 960 to 1920 pixels. Pearson's correlation between mean tracking performance and screen height was 0.141 (p=0.425). Pearson's correlation between mean tracking performance and screen width was -0.360 (p=0.306). Therefore, participants' visual tracking performance is assumed not to have been influenced by the size of the participants' computer screen. However, the participants' browser frame rate was not recorded. Latency may have influenced the speed of objects and disrupted the fluidity of movement during the task. Although participants did not complain about latency issues, it can only be assumed without guarantee that few or no latencies occurred during the experiment. During pre-tests with different computers, browsers and networks, no latency has been experienced. The data required to execute a trial was loaded into the browser's cache before the trial began, which avoided network latency as the virtual players moved. "

The suggested references have been added to the manuscript and the list of references. Also, this reference has been added to the list of references:

- *Franconeri SL, Lin JY, Enns JT, Pylyshyn ZW, Fisher B. Evidence against a speed limit in multiple-object tracking. Psychonomic bulletin & review. 2008;15(4):802–808*

5. **Reviewer 2 point 3e: Please add a few short sentences in the methods section of the manuscript explaining your reasoning in selecting the number of trials.**

Thank you for the comment, the explanation has been added in the methods section of the manuscript (lines 213-217):

"The number of trials (15 per condition) may seem small compared to other investigations on visual tracking expertise [17, 19], but it was intended to encourage participants to remain focused until the end of the experiment. In addition, inter-individual differences in visual tracking performance can appear even with a very low number of trials per condition [18]."

6. **Reviewer 2 point 3f: please include these considerations in the discussion section of the manuscript.**

Thank you for the comment. In this regard, the following paragraph has been added in the discussion section of the manuscript (lines 477-488):

"The ball and the ball carrier are important visual cues that soccer players must focus on to anticipate the outcome of a game situation [24]. North and colleagues (2017) observed that soccer players were more accurate in recognizing familiar soccer situations presented in point light displays when the ball was present than when it was not [53]. In situations with soccer-specific trajectories, the presence of the ball would probably have helped to perceive structured collective behaviors and thus the visual tracking of several players. However, it was difficult to estimate how the presence of the ball would have influenced participants' visual tracking in situations with pseudo-random trajectories. Thus, the ball was removed from the entire experiment because its presence would surely have affected participants' visual tracking performance differently between the two experimental conditions. The

influence of ball presence on the visual tracking in situations with soccer-specific trajectories should be studied in future research."

This reference has been added to the list of references:

- *North, J. S., Hope, E., & Williams, A. M. (2017). The role of verbal instruction and visual guidance in training pattern recognition. Frontiers in psychology, 8, 1473.*

7. Line 531: "Abrupt changes … were observed"

Thank you for this comment, the error has been corrected.
